# A Genome-Wide Association Study Uncovers a Genetic Locus Associated with Thoracic-to-Hip Ratio in Koreans

**DOI:** 10.1371/journal.pone.0145220

**Published:** 2015-12-16

**Authors:** Seongwon Cha, Ah Yeon Park, Changsoo Kang

**Affiliations:** 1 Mibyeong Research Center, Korea Institute of Oriental Medicine, 1672 Yuseongdae-ro, Yuseong-gu, Daejeon 34054, Republic of Korea; 2 Department of Biology and Research Institute of Basic Sciences, College of Natural Sciences, Sungshin Women’s University, Seoul, 01133, Republic of Korea; CAEBi, SPAIN

## Abstract

The thoracic-to-hip circumference ratio (THR) is an anthropometric marker recently described as a predictor of type 2 diabetes. In this study, we performed a genome-wide association study (GWAS) followed by confirmatory analyses to identify genetic markers associated with THR. A total of 7,240 Korean subjects (4,988 for the discovery stage and 2,252 for the confirmatory analyses) were recruited for this study, and genome-wide single nucleotide polymorphism (SNP) genotyping of the initial 4,988 individuals was performed using Affymetrix Human SNP array 5.0. Linear regression analysis was then performed to adjust for the effects of age, sex, and current diabetes medication status on the THR of the study subjects. In the initial discovery stage, there was a statistically nominal association between minor alleles of SNP markers on chromosomes 4, 8, 10, and 12, and THR changes (p < 5.0 × 10^−6^). The subsequent confirmatory analyses of these markers, however, only detected a significant association between two SNPs in the *HECTD4* gene and decreased THRs. Notably, this association was detected in male (rs11066280: p = 1.14 × 10^−2^; rs2074356: p = 1.10 × 10^−2^), but not in female subjects. Meanwhile, the combined results from the two analyses (initial and confirmatory) indicated that minor alleles of these two intronic variants exhibited a significant genome-wide association with decreased THR in the male subjects (n = 3,155; rs11066280: effect size = −0.008624, p = 6.19 × 10^−9^; rs2074356: effect size = −0.008762, p = 1.89 × 10^−8^). Furthermore, minor alleles of these two SNPs exhibited protective effects on patients’ risks for developing type 2 diabetes. In conclusion, we have identified two genetic variations in *HECTD4* that are associated with THR, particularly in men.

## Introduction

Scientific attempts to correlate physical characteristics, such as height and weight, to a specific genetic trait(s) are referred to as genetic anthropometry. Among the anthropometric indices, body mass index (BMI), a general tool for measuring obesity, was suggested as reliable predictor of an individual’s susceptibility to developing type 2 diabetes [1–3]. However, several studies have detected correlations between susceptibility to type 2 diabetes and other anthropometric indices [4–8]. For example, a study of Dutch subjects proposed that the waist-to-hip circumference ratio (WHR) is a better predictor of diabetes susceptibility than BMI [8]. Conversely, in a study of Koreans, the thoracic-to-hip circumference ratio (THR) was indicated as a novel marker for type 2 diabetes, independent of BMI or WHR [4]. This group also detected synergistic effects between the THR and BMI for diabetes risk [4]. Meanwhile, a separate study reported that hip circumference (HC) is inversely associated with the incidence of type 2 diabetes, and that this association was independent of waist circumference (WC) [5].

Although there is some debate regarding which of these anthropometric markers is the best indicator of diabetes risk, the correlations between these different markers and susceptibility to type 2 diabetes could be attributed to the different ethnicities as well as the age and sex distributions of the subjects examined in each study. Indeed, whereas higher HCs were associated with increased susceptibility to diabetes in Chinese individuals, this marker was associated with a decreased risk of diabetes in Caucasian subjects [5,9]. Although there were small discrepancies in each study, analyses of THRs, WHRs, and BMIs consistently suggest that fat distribution in the upper body region (above the hip) is positively associated with increased susceptibility to developing type 2 diabetes. These findings therefore imply that accumulation of visceral fat, rather than that in the hip, might contribute to the development of diabetes by increasing exposure time of the liver to free fatty acids [4,5,9].

Recently, several genome-wide association studies (GWASs) have aimed at identifying genetic markers associated with anthropometric indices; nonetheless, the molecular mechanism by which these anthropometric markers, particularly the THR, affect the amount of an individual’s body fat, and thereby increase diabetes susceptibility, is unclear [10,11]. Furthermore, the genetic traits that affect THRs have yet to be fully elucidated.

In this study, we performed a GWAS to pinpoint genetic loci associated with THRs in a large Korean population. Our results indicate that the genetic markers at *HECTD4* locus are associated with THRs in Korean men.

## Materials and Methods

### Study subjects

Participants included in the GWAS were recruited for community-based cohort studies for the Korean Genome and Epidemiology Study (KoGES) from Ansan and Ansung, Korea, between 2009 and 2012. The KoGES was initiated to identify genetic markers associated with multiple human quantitative traits such as anthropometric indices, lifestyles, and complex diseases, including type 2 diabetes, hypertension, metabolic syndrome, dyslipidemia, and hypertension [12,13].

Individuals with gender inconsistencies, cryptic relatedness, low genotypic call rate, and sample contamination, as well as previous medical history of hypertension, diabetes, dyslipidemia cancer, thyroid disease, coronary artery disease, and female hormone therapy, were excluded for the analysis, however, those who currently take medicine for hypertension, diabetes and dyslipidemia were included in this study. Thus, a total of 4,988 individuals (2,388 males and 2,600 females) were included in the stage 1 (discovery stage) analysis. To ensure that a genotype-phenotype association observed in a stage 1 analysis is not a finding by chance or an artifact due to uncontrolled biases, independent, 2,252 individuals (767 males and 1,485 females) after applying same exclusion criteria described above were recruited from 22 oriental medical clinics for the Korea Constitution Multicenter Study (KCMS) from 2006 to 2012, and were included in the stage 2 (confirmatory) analysis. All subjects provided written, informed consent to participate in this study, and the study was approved by the Institutional Review Boards of the Korea Centers for Disease Control and Prevention (for KoGES) and the Korea Institute of Oriental Medicine (for KCMS).

The anthropometric dimensions of each subject were measured horizontally using a tapeline by trained operators following the previously described standardized procedure [11]. Thoracic (ThC), waist (WC), and hip (HC) circumferences were measured between inspiration and expiration while subjects stood erect; ThC, WC, and HC were measured at the levels of the 7^th^–8^th^ costochondral junctions, umbilicus, and upper margin of the pubis, respectively. THR and WHR were calculated by dividing respective ThC and WC values by the HC value. Individuals with hyperglycemia, impaired fasting glucose (IFG), and diabetes mellitus (DM) were defined as subjects exhibiting fasting blood glucose levels ≥ 110 mg/dL, ≥ 110 mg/dL and < 126 mg/dL, and ≥ 126 mg/dL, respectively, or as individuals that were prescribed diabetes medication.

### SNP Genotyping

Genome-wide SNP genotyping of the KoGES subjects was performed using the Affymetrix Human SNP array 5.0 (Affymetrix, Santa Clara, CA), as previously described [13]. Of the 500,568 SNPs examined, those exhibiting high missing call rates (>5%), low minor allele frequencies (<0.05), or significant deviations from the Hardy-Weinberg equilibrium (HWE; p < 0.0001) were excluded for quality control. After these analyses, the remaining 310,746 SNPs were subjected to further analyses. The seven variants (rs11066280, rs2074356, rs12229654, rs11201882, rs6531296, rs12114850, and rs6852847) that exhibited statistically nominal associations with THRs in the initial discovery stage were assessed in the KCMS subjects (n = 2,252) by TaqMan® assay analysis or by melting analysis of an unlabeled oligonucleotide probe (UOP) applied during PCR [14]. All seven variants were within the HWE in the KCMS population (p > 0.05).

### Statistical analysis

During the discovery stage, GWAS was performed to identify variants associated with the THR by linear regression analysis in an additive model using PLINK version 1.07 (http://pngu.mgh.harvard.edu/purcell/plink/) [15]. Age, sex, and current diabetes medication status were employed as covariates to adjust for their effects on the THR. Quantile-quantile plots for THRs were constructed using the distribution of observed p-values against the theoretical distribution of expected p-values. The genomic control inflation factors (λ) for GWAS were checked for potential p-value inflation. Manhattan plots for the THRs of males, females, and the combined sample groups were generated using R version 3.0.2 software (http://www.r-project.org/), and the regional plot, with a 1-megabase (Mb) window centered at the variant with strongest association on chromosome 12, was constructed using the web-based LocusZoom tool [16].

In the replication study, linear regression analyses of the THRs were performed by adjusting for the effects of age, sex, and current diabetes medication status on the KCMS subjects (n = 2,252). Associations between THR-associated variants and diabetes-related traits, such as hyperglycemia, IFG, and DM, were estimated by logistic regression after controlling for age and sex. Sex interaction of THR-associated variants was assessed by introducing an interaction term in the regression analyses for THR and diabetes diabetes-related traits. Regression analyses of individual SNPs were performed using R software, and Chi-squared tests were used to determine whether the variants deviated from the HWE. Linkage disequilibrium (Lewontin’s *D′* = D/D_max_ and r^2^) was obtained using Haploview version 4.2 (Daly Lab at the Broad Institute, Cambridge, MA, USA) [17]. The association results from the KoGES and KCMS populations were combined using the Comprehensive Meta-Analysis program, version 2.0 (Biostat, Englewood, NJ, USA), with a random effect model using the DerSimonian and Laird method [18]. Genome-wide significance at the Bonferroni-corrected level (0.05/311,944 SNPs) and nominal significance in the GWAS (stage 1) were defined as p < 1.6 × 10^−7^ and p < 5.0 × 10^−6^, respectively, and we regarded p-value of 0.05 as significant level in the confirmatory (stage 2) analysis.

## Results

We analyzed the effects of common variants on the THRs of two separate Korean populations, as follows: GWAS of the KoGES population, comprising 4,988 individuals (discovery stage: stage 1), and confirmatory analysis using the KCMS population, comprising 2,252 individuals (confirmation stage: stage 2). The baseline characteristics of these two groups of subjects, including traits related to cardiometabolic risk, are presented in Table 1. The mean age of the individuals in the KoGES population (60.3 ± 8.6) was higher than that in the KCMS population (46.1 ± 15.7). Likewise, the proportion of males in the KoGES group was higher (48%) than in the KCMS group (34%). Therefore, we performed a regression analysis to exclude the effects of both age and sex on the THR trait (Table 1).

**Table 1:** Baseline characteristics of the study subjects.

Characteristics	KoGES			KCMS		
	All	Male	Female	All	Male	Female
	(n = 4,988)	(n = 2,388)	(n = 2,600)	(n = 2,252)	(n = 767)	(n = 1,485)
**Age (yrs)**	60.3 ± 8.6	59.8 ± 8.4	60.9 ± 8.6	46.1 ± 15.7	46.0 ± 16.2	46.2 ± 15.5
**Body mass index (kg/m** ^**2**^ **)**	24.4 ± 3.1	24.2 ± 2.9	24.6 ± 3.2	23.1 ± 3.3	23.7 ± 3.2	22.7 ± 3.2
**Waist circumference (cm)**	86.3 ± 8.4	86.8 ± 7.9	85.8 ± 8.9	82.8 ± 9.8	86.2 ± 8.9	81.0 ± 9.7
**Thoracic circumferenc (cm)**	84.2 ± 7.8	87.6 ± 6.3.	81.0 ± 7.8	80.5 ± 9.5	86.4 ± 8.0	77.4 ± 8.8
**Waist-to-hip ratio**	0.93 ± 0.063	0.94 ± 0.054	0.92 ± 0.069	0.89 ± 0.073	0.91 ± 0.061	0.87 ± 0.075
**Thoracic-to-hip ratio**	0.91 ± 0.065	0.95 ± 0.044	0.87 ± 0.06	0.86 ± 0.078	0.92 ± 0.062	0.84 ± 0.070
**Systolic blood pressure (mmHg)**	119.8 ± 16.8	120.4 ± 15.7	119.3 ± 17.8	117.5 ± 14.8	121.0 ± 14.0	115.7 ± 14.8
**Diastolic blood pressure (mmHg)**	77.6 ± 10.3	79.1 ± 10.0	76.3 ± 10.4	75.4 ± 10.6	77.9 ± 10.7	74.2 ± 10.3
**HDL cholesterol (mg/dL)**	45.9 ± 12.3	44.7 ± 11.9	47.1 ± 12.5	48.5 ± 12.4	43.5 ± 10.8	51.1 ± 12.4
**Triglyceride (mg/dL)**	142.3 ± 97.1	148.8 ± 106	136.4 ± 87.7	116.0 ± 74.9	138.3 ± 84.3	104.6 ± 66.7
**Fasting blood glucose (mg/dL)**	100.9 ± 25.4	102.2 ± 23.7	96.7 ± 26.7	96.4 ± 25.2	99.2 ± 26.0	95.0 ± 24.7

Values are presented as means ± standard deviations.

Abbreviations: KoGES, Korean Genome and Epidemiology Study; KCMS, Korea Constitution Multicenter Study

### Common variants associated with THR

**Fig 1:**
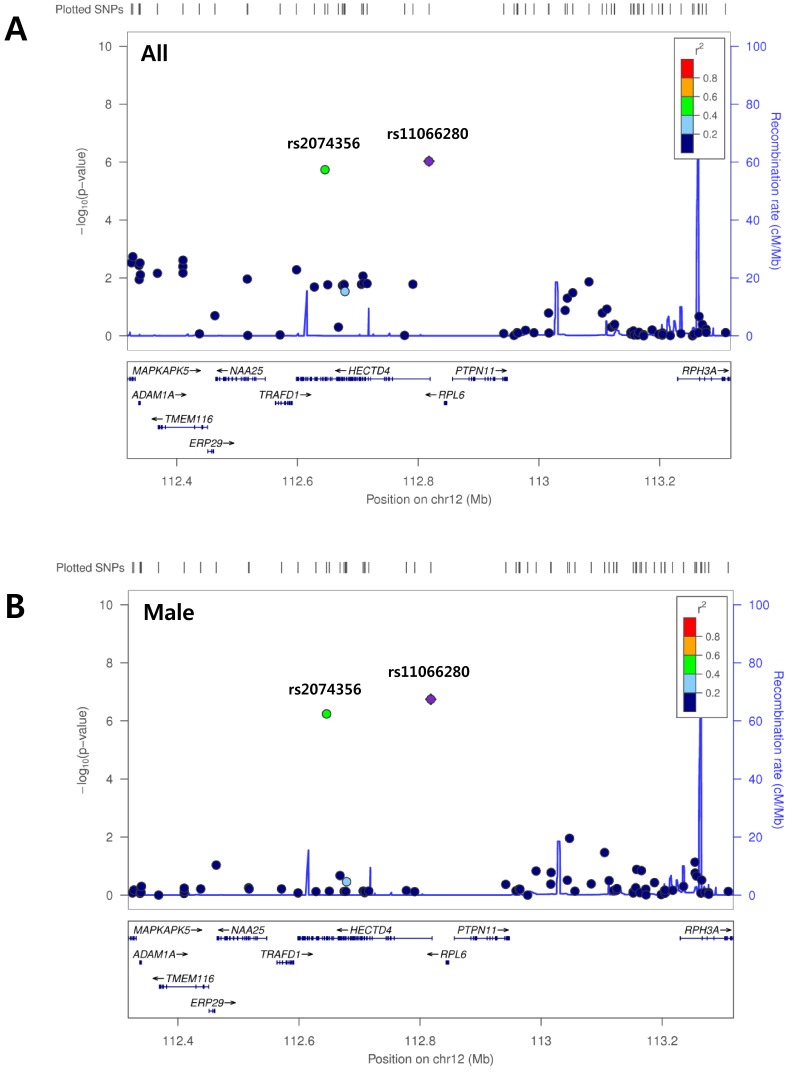
Magnification of association plots of the *HECTD4* region on chromosome 12. The image depicts a 1-Mb (megabase) chromosomal region containing variant rs11066280 at its center. Panel A represents the genome-wide association study (GWAS) results for all the subjects (both male and female) and panel B represents the GWAS results for the male subjects.

We performed a GWAS to identify genetic variants associated with THRs in all subjects of the KoGES population, as well as in males and females, respectively (stage 1). Quantile-quantile plots indicated deviations only in the extreme tail probabilities between the distributions of expected and observed p-values (λ = 1.026 for all subjects; λ = 1.024 for males; and λ = 1.011 for females); thus, the effects of population stratification were considered negligible (S1 Fig). Meanwhile, the GWAS analysis of the entire population detected that minor alleles of the two intronic SNPs in the *HECTD4* (HECT domain containing E3 ubiquitin protein ligase 4) gene, located at chromosome 12q24.13 exhibited nominal association with decreased THRs, as shown in Table 2 and S2 Fig (rs11066280, effect size = −0.006521, standard error (SE) = 0.001328, p = 9.36 × 10^−7^; and rs2074356, effect size = −0.006700, SE = 0.001403, p = 1.84 × 10^−6^). Notably, the significance values for the association of these variants with the THR were higher in the analysis of the male subjects (p = 1.80 × 10^−7^ and 5.76 × 10^−7^, respectively) (Table 2), as depicted in the regional plot containing a 1-Mb genomic region centered at the SNP showing the strongest association (Fig 1). Meanwhile, minor alleles of the two variants located on chromosomes 8 and 4 were found to be nominally associated with decreased THR during analysis of the female population (Table 2).

**Table 2:** Linear regression analysis for the THR.

Variant	Gene (Allele, Chr)	Subjects	MAF	Stage 1				Stage 2				Combined	
				n	Effect size (SE)	P	P_sex_	n	Effect size (SE)	P	P_sex_	Effect size (SE)	P
rs11066280	*HECTD4* (T>A, 12)	All	0.17	4988	−0.006521 (0.001328)	9.36×10^−7^	0.0870	2229	−0.003232 (0.002354)	0.170	0.122	−0.005447 (0.001543)	4.17×10^−4^
		Male	0.18	2388	−0.008465 (0.001627)	1.80×10^−7^	-	758	−0.009476 (0.003737)	1.14×10^−2^	-	−0.008624 (0.001484)	6.19×10^−9^
		Female	0.17	2600	−0.004384 (0.002031)	3.10×10^−2^	-	1471	0.0004263 (0.002982)	0.886	-	−0.002474 (0.002354)	0.293
rs2074356	*HECTD4* (C>T, 12)	All	0.16	4987	−0.006700 (0.001403)	1.84×10^−6^	0.139	2236	−0.002208 (0.002326)	0.343	0.045	−0.004840 (0.002212)	2.86×10^−2^
		Male	0.16	2387	−0.008663 (0.001728)	5.76×10^−7^	-	763	−0.009193 (0.003607)	1.10×10^−2^	-	−0.008762 (0.001558)	1.89×10^−8^
		Female	0.16	2600	−0.004808 (0.002123)	2.37×10^−2^	-	1473	0.002668 (0.002989)	0.372	-	−0.001366 (0.003726)	0.714
rs12229654	*CCDC63* (T>G, 12)	All	0.14	4987	−0.006750 (0.001440)	2.85×10^−6^	0.992	2229	−0.003153 (0.002538)	0.214	0.376	−0.005558 (0.001694)	1.04×10^−3^
		Male	0.14	2387	−0.006703 (0.001775)	1.63×10^−4^	-	759	−0.003610 (0.004231)	0.394	-	−0.006240 (0.001637)	1.38×10^−4^
		Female	0.14	2600	−0.006724 (0.002182)	2.08×10^−3^	-	1470	−0.002701 (0.003139)	0.390	-	−0.005345 (0.001909)	5.12×10^−3^
rs11201882	*GRID1* (C>T, 10)	All	0.48	4987	−0.004619 (0.001010)	4.89×10^−6^	0.301	2221	0.002075 (0.001796)	0.248	0.660	−0.001435 (0.003345)	0.668
		Male	0.48	2387	−0.003495 (0.001249)	5.19×10^−3^	-	759	0.001952 (0.002927)	0.505	-	−0.001415 (0.002646)	0.593
		Female	0.47	2600	−0.005181 (0.001524)	6.88×10^−4^	-	1462	0.001137 (0.002257)	0.615	-	−0.002242 (0.003151)	0.477
rs6531296	*RPL31P31* (A>G, 4)	All	0.28	4942	0.002250 (0.001114)	4.34×10^−2^	2.39×10^−4^	2227	0.001307 (0.001981)	0.510	0.169	0.002024 (0.009709)	3.71×10^−2^
		Male	0.28	2363	0.006508 (0.001390)	3.01×10^−6^	-	758	0.004278 (0.003360)	0.203	-	0.006182 (0.001284)	1.49×10^−6^
		Female	0.29	2579	−0.001726 (0.001660)	0.299	-	1469	0.0002164 (0.002430)	0.929	-	−0.001108 (0.001371)	0.419
rs12114850	*KHDRBS3* (C>G, 8)	All	0.12	4988	−0.005276 (0.001525)	5.45×10^−4^	6.28×10^−6^	2236	0.002934 (0.002727)	0.282	0.171	−0.001485 (0.004091)	0.717
		Male	0.13	2388	0.001967 (0.001864)	0.292	-	763	−0.003070 (0.004412)	0.487	-	0.001036 (0.001956)	0.596
		Female	0.13	2600	−0.01206 (0.002321)	2.17×10^−7^	-	1473	0.005619 (0.003423)	0.101	-	−0.003399 (0.008838)	0.701
rs6852847	*TMEM248P1* (A>T, 4)	All	0.26	4826	−0.004406 (0.001171)	1.69×10^−4^	9.82×10^−4^	2212	0.001832 (0.002014)	0.363	0.443	−0.001502 (0.003111)	0.629
		Male	0.26	2304	−0.0007494 (0.001456)	0.607	-	753	0.002914 (0.003254)	0.371	-	0.00007338 (0.001421)	0.959
		Female	0.26	2522	−0.008553 (0.001755)	1.17×10^−6^	-	1459	0.001295 (0.002530)	0.609	-	−0.003798 (0.004921)	0.440

Abbreviations: THR, thoracic-to-hip circumference ratio; Chr, chromosome; MAF, minor allele frequency; SE, standard error; HECTD4, HECT domain containing E3 ubiquitin protein ligase 4; CCDC63, coiled-coil domain containing 63; GRID1, glutamate receptor, ionotropic, delta 1; RPL31P31, ribosomal protein L31 pseudogene 31; KHDRBS3, KH domain containing, RNA binding, signal transduction associated 3; TMEM248P1, transmembrane protein 248 pseudogene 1. Effect size (**β**) presenting THR changes per minor allele, SE, and p-value (P) were calculated by linear regression analysis with adjustment for age, current diabetes medication status, and/or sex in an additive genetic model: (stage 1) genome-wide association analysis of the KoGES population; (stage 2) confirmatory analysis using the KCMS population. P_sex_ was assessed by introducing an interaction term between sex and variant genotype in the regression analysis.

To confirm the correlations detected between the variants and the THRs, we performed a confirmatory analysis (stage 2) using the KCMS population. Consistent with the results of the stage 1 analysis, there was a nominal correlation between the minor alleles of the two *HECTD4* variants, rs11066280 and rs2074356, and decreased THRs in male subjects (rs11066280: effect size = −0.009476, SE = 0.003737, p = 1.14 × 10^−2^; rs2074356: effect size = −0.009193, SE = 0.003607, p = 1.10 × 10^−2^) (Table 2). Furthermore, there was a significant association between these two SNPs and THRs (genome-wide significance) in male subjects after combining the results from both the stage 1 and 2 analyses (rs11066280: effect size = −0.008624, SE = 0.001484, p = 6.19 × 10^−9^; rs2074356: effect size = −0.008762, SE = 0.001558, p = 1.89 × 10^−8^) (Table 2). The two significant SNPs in male subjects did not show sex interaction in stage 1 population, whereas only the rs2074356 presented association in the level of p < 0.05 in stage 2 population (Table 2).

### Effects of THR-associated variants on diabetic risk

A previous study demonstrated a correlation between lower THRs and decreased diabetic risks [4]. As a result, we assessed the effects of the two THR-associated *HECTD4* variants on diabetes-related traits, such as hyperglycemia, IFG, and DM, in the entire study group, as well as in male and female subjects, respectively. Minor alleles of the two SNPs were found to exert protective effects on all three diabetic-related traits. After subgrouping by gender, however, only the associations between rs2074356 and both hyperglycemia and IFG remained significant and enriched in the male population (rs2074356 in men: odds ratio (OR) = 0.742, p = 5.27 × 10^−3^ for hyperglycemia; OR = 0.622, p = 7.95 × 10^−3^ for IFG) (Table 3). These findings indicate that the minor alleles of the *HECTD4* variants identified in this study, particularly the rs2074356 SNP, have protective effects in developing diabetes in males.

**Table 3:** Combined logistic regression analysis of THR-association variants with hyperglycemia, impaired fasting glucose, and diabetes mellitus.

Variant	Gene (Allele, Chr)	MAF	Hyperglycemia			Impaired fasting glucose			Diabetes mellitus		
			OR (SE)	P	P_sex_	OR (SE)	P	P_sex_	OR (SE)	P	P_sex_
**All**											
**n (case/control)**			1374/5862			838/5862			747/5862		
**rs11066280**	*HECTD4* (T>A, 12)	0.17	0.8088 (0.05969)	3.78×10^−4^	0.380	0.7708 (0.07426)	4.55×10^−4^	0.335	0.8060 (0.09039)	1.70×10^−2^	0.599
**rs2074356**	*HECTD4* (T>A, 12)	0.16	0.8007 (0.06250)	3.74×10^−4^	0.394	0.7464 (0.08197)	3.59×10^−4^	0.330	0.8189 (0.8087)	1.35×10^−2^	0.772
**Male**											
**n (case/control)**			710/2444			438/2444			375/2444		
**rs11066280**	*HECTD4* (T>A, 12)	0.18	0.6436 (0.03031)	0.146	-	0.5641 (0.3578)	0.11	-	0.7337 (0.2197)	0.159	-
**rs2074356**	*HECTD4* (T>A, 12)	0.16	0.7423 (0.001068)	5.27×10^−3^	-	0.6218 (0.1790)	7.95×10^−3^	-	0.8100 (0.1132)	0.063	-
**Female**											
**n (case/control)**			664/3418			400/3418			372/3418		
**rs11066280**	*HECTD4* (T>A, 12)	0.17	0.8913 (0.1158)	0.32	-	0.9679 (0.2427)	0.893	-	0.8319 (0.1104)	0.095	-
**rs2074356**	*HECTD4* (T>A, 12)	0.16	0.9251 (0.2005)	0.698	-	0.9490 (0.3008)	0.862	-	0.8378 (0.1151)	0.124	-

Abbreviations: THR, thoracic-to-hip circumference ratio; Chr, chromosome; MAF, minor allele frequency; OR, odds ratio; SE, standard error; *HECTD4*, HECT domain containing E3 ubiquitin protein ligase 4. OR, SE, and p-value (P) were calculated by logistic regression analysis with adjustment for age and/or sex in an additive genetic model: combined analysis of association results from both the KoGES and KCMS populations. P_sex_ (combined from two populations) was assessed by introducing an interaction term between sex and variant genotype in the regression analysis.

## Discussion

The THR is one of several anthropometric markers used to predict an individual’s risks for developing type 2 diabetes [4]. In this study, we performed a two-staged analysis, consisting of a GWAS (discovery) and a confirmatory analysis, to uncover genetic markers associated with the THR. Our analyses suggest that minor alleles of the two *HECTD4* variants, rs11066280 and rs2074356, which localized to IVS1 + 1973 and IVS51 + 22, respectively (NM_001109662), were associated with reduced THRs in Korean men. Interestingly, theses THR-associated variants were previously reported to exert pleiotropic effects on anthropometric and metabolic traits, including WHR, high-density lipoprotein cholesterol levels, hepatic traits, and diastolic blood pressure, in East Asians [11,19–21]. Furthermore, the rs11066280 SNP was reported to be associated with systolic and diastolic blood pressure variations in 19,608 subjects from East Asia [20]. Lastly, while the results of a separate GWAS of Asian subjects suggested that rs2074356 is a genetic factor associated with WHR, an association between this variant and THR was not described [11]. In our study, we detected only weak association signal between the rs2074356 SNP and the WHR of our study subjects (particularly men), compared to those measured between this SNP and THRs in the KoGES subjects, as shown in S1 Table (on WHR: effect size = −0.007820, p = 2.46 × 10^−4^; on THR: effect size = −0.008663, p = 5.76 × 10^−7^). Moreover, this association was not replicated in the KCMS subjects (p = 0.126), and diminished associations with WHR were detected for the other THR-associated SNPs in the *HECTD4* region (S1 Table). We therefore propose that the *HECTD4* region harbors a genetic factor that strongly influences THR but not WHR.

While our sex-stratified analysis detected only a significant association between the *HECTD4* variants and the THR of male subjects, it is unclear why there was no positive association with the THR of the female subjects (Table 2). One possible explanation for this phenomenon, however, is that females may undergo more dramatic changes of anthropometric indices than men, particularly in the hip area during pregnancy and after childbirth. These changes may therefore hinder the detection of significant associations with the THRs of females.

While the mechanism by which the *HECTD4* region may influence susceptibility to type 2 diabetes, beyond increasing an individual’s THR, is poorly understood, *HECTD4* is known as a gene that is pleiotropic for obesity/adiposity and inflammation [21], which could contribute to this process. Furthermore, a previous study using a mouse obesity model revealed that increased levels of adipose tissue, particularly in the liver, promoted chronic tissue inflammation and subsequently lead to the development of insulin resistance [22]. Thus, it is conceivable that obesity at the upper trunk region of the human body, as characterized by high THRs, may increase susceptibility to type 2 diabetes.

One limitation of our study is that we were unable to evaluate the functional effects of the THR-associated SNPs rs11066280 and rs2074356. Therefore, we cannot exclude the possibility that another variant(s) in linkage disequilibrium with these two SNPs may alter the expression levels of the *HECTD4* gene or the activity of the protein product. As such, our findings warrant further functional study to support the observed correlation between variants in the *HECTD4* region and THR.

In summary, we present the first findings demonstrating that the *HECTD4* region comprises a genetic locus that is linked to THRs in Koreans. Furthermore, our results suggest that obesity at the upper trunk, rather than at the waist, of the human body may result in inflammation that can lead to the development of type 2 diabetes.

## Supporting Information

S1 Fig Quantile-quantile plots of the THRs. (PPTX)

S2 Fig Genome-wide association results for the thoracic-to-hip circumference ratios (THRs) in the Stage 1 analysis. Manhattan plots of the p-values (-log_10_(p)) show the variants that were associated with THRs in the entire population (A) and in the male (B) and female (C) subjects. Red lines denote a p-value of 5.0 × 10^−6^.
(PPTX)

S1 Table Linear regression analysis of WHRs. (DOCX)
