# Supplementary figures and images for: A Genome-Wide Association Study Uncovers a Genetic Locus Associated with Thoracic-to-Hip Ratio in Koreans
Source: PLoS One. 2015 Dec 16;10(12):e0145220. doi: 10.1371/journal.pone.0145220 (PMC4686062; doi:10.1371/journal.pone.0145220)

## Slide 1
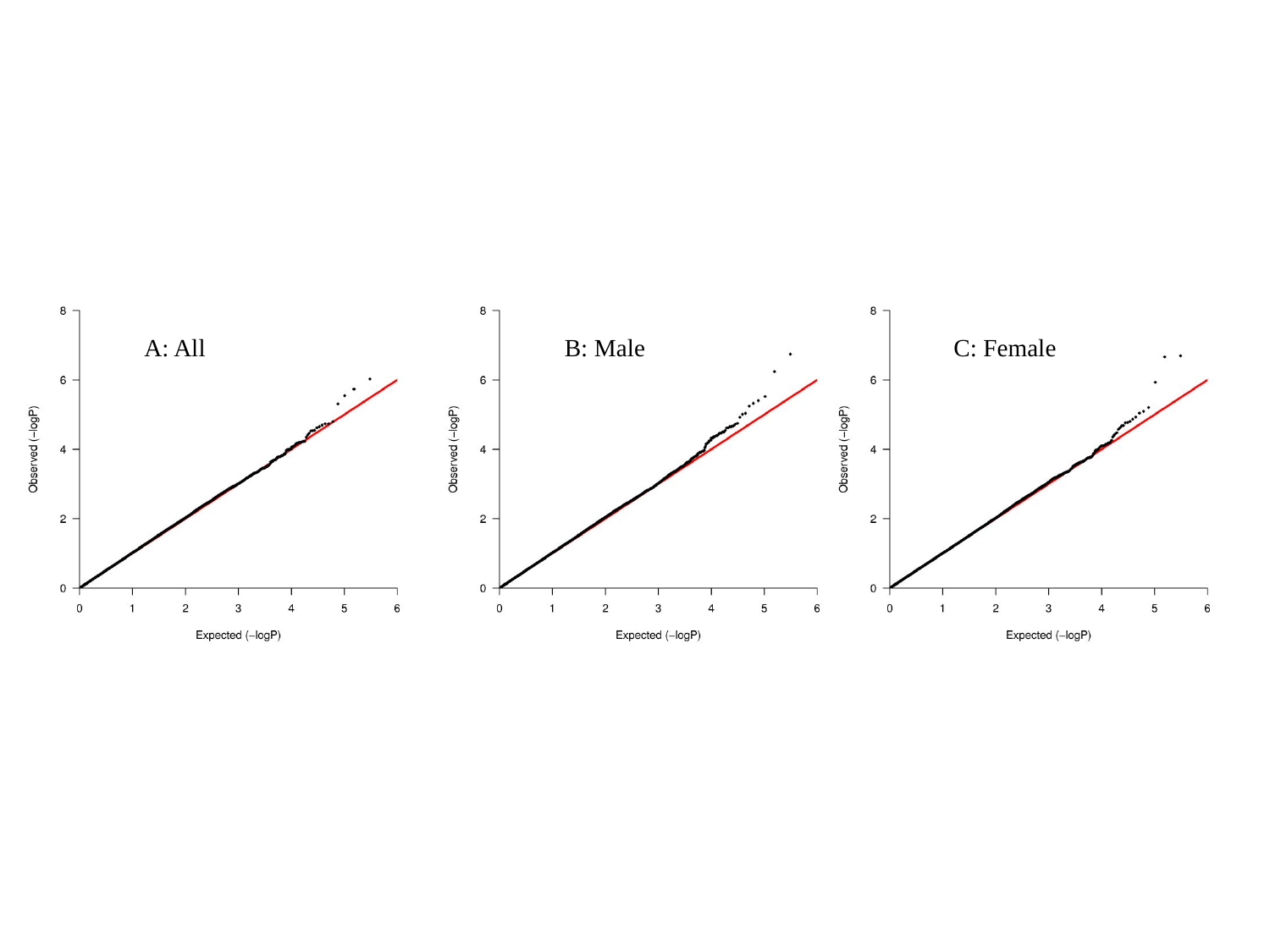

A: All
B: Male
C: Female

Supplement: S1 Fig — (PPTX) [file pone.0145220.s001.pptx]

## Slide 1
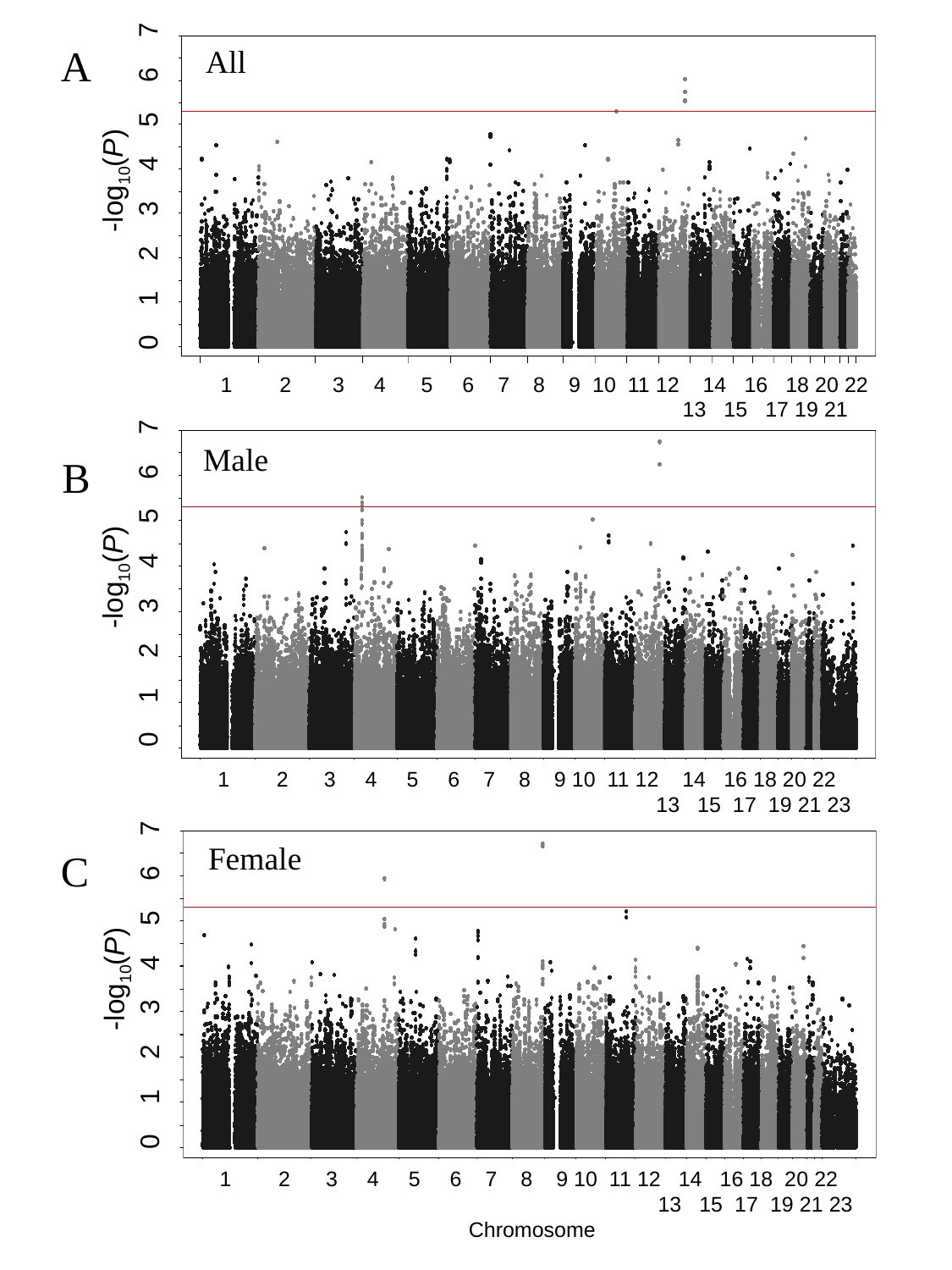

-log10(P)
0 1 2 3 4 5 6 7
A
All
 1 2 3 4 5 6 7 8 9 10 11 12 14 16 18 20 22
 13 15 17 19 21
-log10(P)
0 1 2 3 4 5 6 7
Male
B
 1 2 3 4 5 6 7 8 9 10 11 12 14 16 18 20 22
 13 15 17 19 21 23
-log10(P)
0 1 2 3 4 5 6 7
Female
C
 1 2 3 4 5 6 7 8 9 10 11 12 14 16 18 20 22
 13 15 17 19 21 23
Chromosome

Supplement: S2 Fig — Manhattan plots of the p-values (-log10(p)) show the variants that were associated with THRs in the entire population (A) and in the male (B) and female (C) subjects. Red lines denote a p-value of 5.0 × 10^−6^. (PPTX) [file pone.0145220.s002.pptx]
