# Supplementary material for: A Genome-Wide Association Study Uncovers a Genetic Locus Associated with Thoracic-to-Hip Ratio in Koreans
Source: PLoS One. 2015 Dec 16;10(12):e0145220. doi: 10.1371/journal.pone.0145220 (PMC4686062; doi:10.1371/journal.pone.0145220)
Supplement: S1 Table — (DOCX) [file pone.0145220.s003.docx]

**S1 Table.** **Linear regression analysis for WHR.**

|  | **Variant** | **Chr** | **Gene** | **Allele** | **MAF** | **KoGES** | | | **KCMS** | | | **Combined** | |
| --- | --- | --- | --- | --- | --- | --- | --- | --- | --- | --- | --- | --- | --- |
|  |  |  |  |  |  | **n** | **Effect size (SE)** | **p** | **n** | **Effect size (SE)** | **p** | **Effect size (SE)** | **p** |
| **All** | rs11066280 | 12 | *HECTD4* | T>A | 0.17 | 4988 | −0.005570 (0.001560) | 3.59ⅹ10^-4^ | 2229 | −0.003973 (0.002460) | 0.106 | −0.005111 (0.001317) | 1.05ⅹ10^-4^ |
|  | rs2074356 | 12 | *HECTD4* | C>T | 0.16 | 4987 | −0.005811 (0.001647) | 4.23ⅹ10^-4^ | 2236 | −0.003761 (0.002430) | 0.122 | −0.005165 (0.001363) | 1.51ⅹ10^-4^ |
|  | rs12229654 | 12 | *CCDC63* | T>G | 0.14 | 4987 | −0.006686 (0.001691) | 7.36ⅹ10^-5^ | 2229 | −0.004212 (0.002652) | 0.112 | −0.005970 (0.001426) | 2.82ⅹ10^-6^ |
|  | rs11201882 | 10 | *GRID1* | C>T | 0.48 | 4987 | −0.003049 (0.001186) | 1.02ⅹ10^-2^ | 2221 | 0.004387 (0.001876) | 1.94ⅹ10^-2^ | 0.0005275 (0.003717) | 0.887 |
| **Male** | rs11066280 | 12 | *HECTD4* | T>A | 0.18 | 2388 | −0.007188 (0.001995) | 3.21ⅹ10^-4^ | 758 | −0.008570 (0.003710) | 2.12ⅹ10^-2^ | −0.007498 (0.001757) | 1.98ⅹ10^-5^ |
|  | rs2074356 | 12 | *HECTD4* | C>T | 0.16 | 2387 | −0.007820 (0.002130) | 2.46ⅹ10^-4^ | 763 | −0.005496 (0.003590) | 0.126 | −0.007215 (0.001832) | 8.20ⅹ10^-5^ |
|  | rs6531296 | 4 | *RPL31P31* | A>G | 0.28 | 2363 | 0.004011 (0.001717) | 1.96ⅹ10^-2^ | 758 | 0.004028 (0.003332) | 0.227 | 0.004015 (0.001526) | 8.53ⅹ10^-3^ |
| **Female** | rs12114850 | 8 | *KHDRBS3* | C>G | 0.13 | 2600 | −0.01081 (0.002632) | 4.10ⅹ10^-5^ | 1473 | 0.006705 (0.003650) | 0.0664 | −0.002235 (0.008756) | 0.799 |
|  | rs6852847 | 4 | *TMEM248P1* | A>T | 0.26 | 2522 | −0.005590 (0.001996) | 5.13ⅹ10^-3^ | 1459 | 0.001121 (0.002706) | 0.679 | −0.002483 (0.003346) | 0.458 |

Abbreviations: WHR, waist-to-hip circumference ratio; Chr, chromosome; MAF, minor allele frequency; SE, standard error; HECTD4, HECT domain containing E3 ubiquitin protein ligase 4; CCDC63, coiled-coil domain containing 63; GRID1, glutamate receptor, ionotropic, delta 1; RPL31P31, ribosomal protein L31 pseudogene 31; KHDRBS3, KH domain containing, RNA binding, signal transduction associated 3; TMEM248P1, transmembrane protein 248 pseudogene 1

Linear regression analysis with adjustment for age, current diabetes medication status, and/or sex in an additive genetic model
